# Supplementary material for: Identification of Pinocembrin as an Anti-Glycation Agent and α-Glucosidase Inhibitor from Fingerroot (Boesenbergia rotunda): The Tentative Structure–Activity Relationship towards MG-Trapping Activity
Source: Molecules. 2018 Dec 19;23(12):3365. doi: 10.3390/molecules23123365 (PMC6321453; doi:10.3390/molecules23123365)
Supplement: Supplementary file 1 [file molecules-23-03365-s001.pdf]

Supplementary Material

Identification of Pinocembrin as preventive and anti-glycation and anti-diabetic agent from fingerroot (*Boesenbergia rotunda*): The tentative structure-activity relationship towards MG-trapping activity

Thammatee Potipiranun [1,2], Sirichai Adisakwattana [3], Wisuttaya Worawalai [2], Rico Ramadhan [2] and Preecha Phuwapraisirisan [2,4,*]

[1] Program of Biotechnology, Faculty of Science, Chulalongkorn University, Bangkok 10330, Thailand

[2] Center of Excellence in Natural Products, Department of Chemistry, Faculty of Science, Chulalongkorn University, Bangkok 10330, Thailand

[3] Department of Nutrition and Dietetics, Faculty of Allied Health Sciences, Chulalongkorn University, Bangkok 10330, Thailand

* Correspondence: Preecha.p@chula.ac.th; Tel 662-2187624; Fax 662-2187598

Lis of supplementary information

Figure S1. ^{1}H NMR spectrum (400 MHz, CD_3OD) of pinocembrin (**1**)

Figure S2. ^{1}H NMR spectrum (400 MHz, $CDCl_3$) of pinostrobrin (**2**)

Figure S3. ^{1}H NMR spectrum (400 MHz, CD_3OD) of alpinetin (**3**)

Figure S4. ^{1}H NMR spectrum (400 MHz, CD_3OD) of cardamomin (**4**)

Figure S5. ^{13}C NMR spectrum (400 MHz, CD_3OD) of cardamomin (**4**)

Figure S6. ^{1}H NMR spectrum (400 MHz, $CDCl_3$) of boesenbergin B (**5**)

Figure S7. ^{13}C NMR spectrum (400 MHz, $CDCl_3$) of boesenbergin B (**5**)

Figure S8. ^{1}H NMR spectrum (400 MHz, $CDCl_3$) of panduratin A (**6**)

Figure S9. ^{13}C NMR spectrum (400 MHz, $CDCl_3$) of panduratin A (**6**)

Figure S10. ^{1}H NMR spectrum (400 MHz, $CDCl_3$) of isopanduratin A (**7**)

Figure S11. ^{1}H NMR spectrum (400 MHz, $CDCl_3$) of demethoxyyangonin (**8**)

Figure S12. ^{13}C NMR spectrum (400 MHz, $CDCl_3$) of demethoxyyangonin (**8**)

Figure S13. Inhibition plot of pinicembrin against rat intestinal maltase.

Figure S14. Inhibition plot of pinicembrin against rat intestinal sucrase.

Figure S15. The HPLC chromatogram of MG (0.01–1mM) after reaction with AG (0.1 mM) and pinocembrin (0.1 mM) for 24 h. MG was detected as 2methylquinoxaline (2-MQ) after derivatization using *o*- phenylenediamine (OPD). 5-Methylquinoxaline (5-MQ) was used as the internal standard.

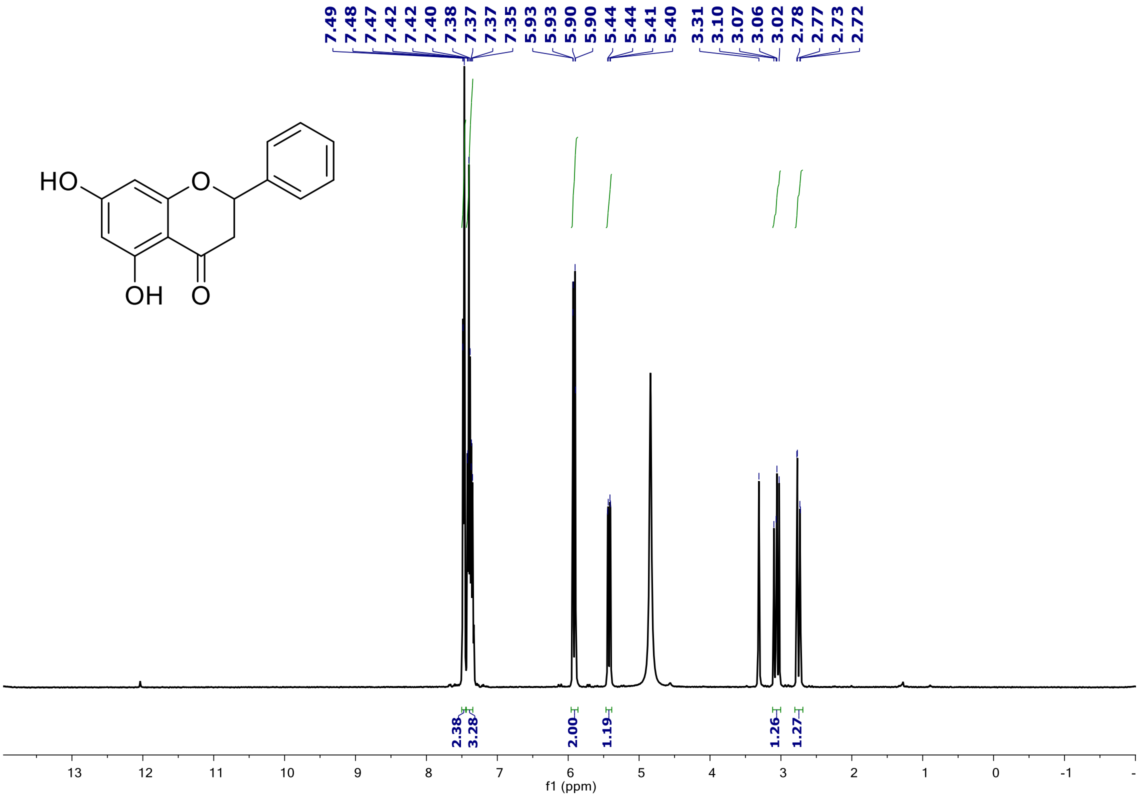

Figure S1. ^{1}H NMR spectrum (400 MHz, CD_3OD) of pinocembrin (**1**)

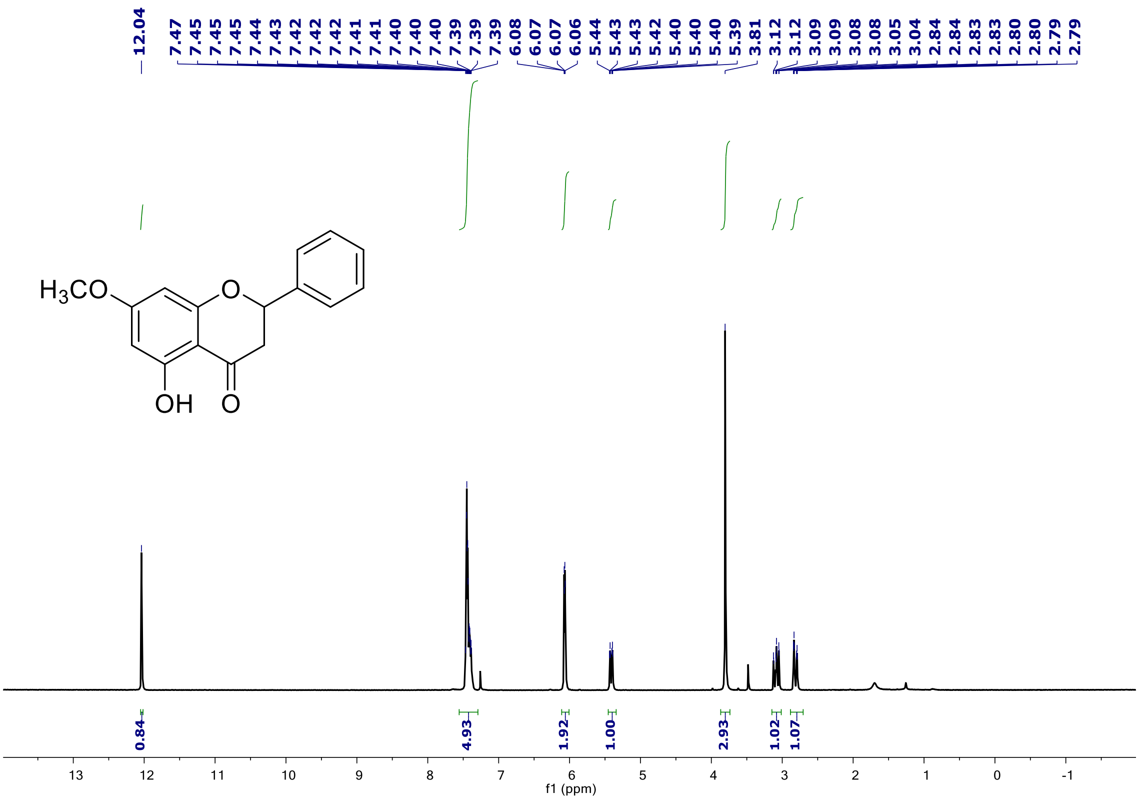

Figure S2. ^{1}H NMR spectrum (400 MHz, $CDCl_3$) of pinostrobin (**2**)

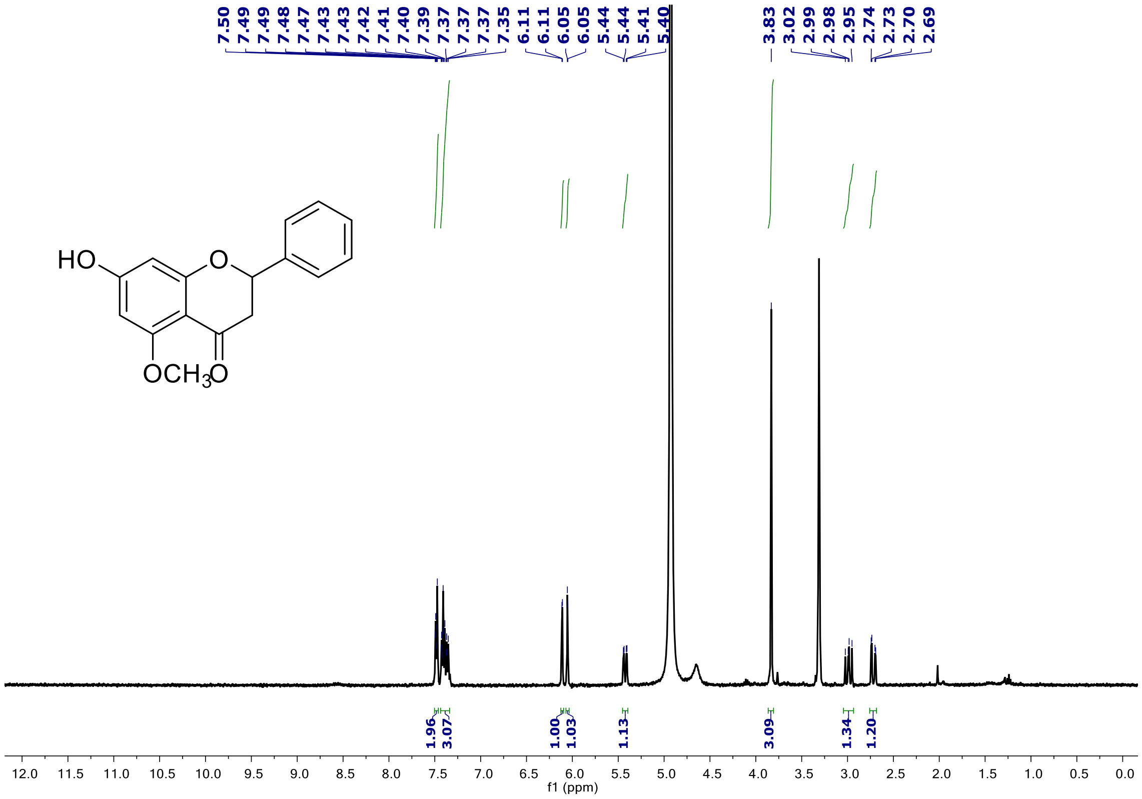

Figure S3. ^{1}H NMR spectrum (400 MHz, CD_3OD) of alpinetin (**3**)

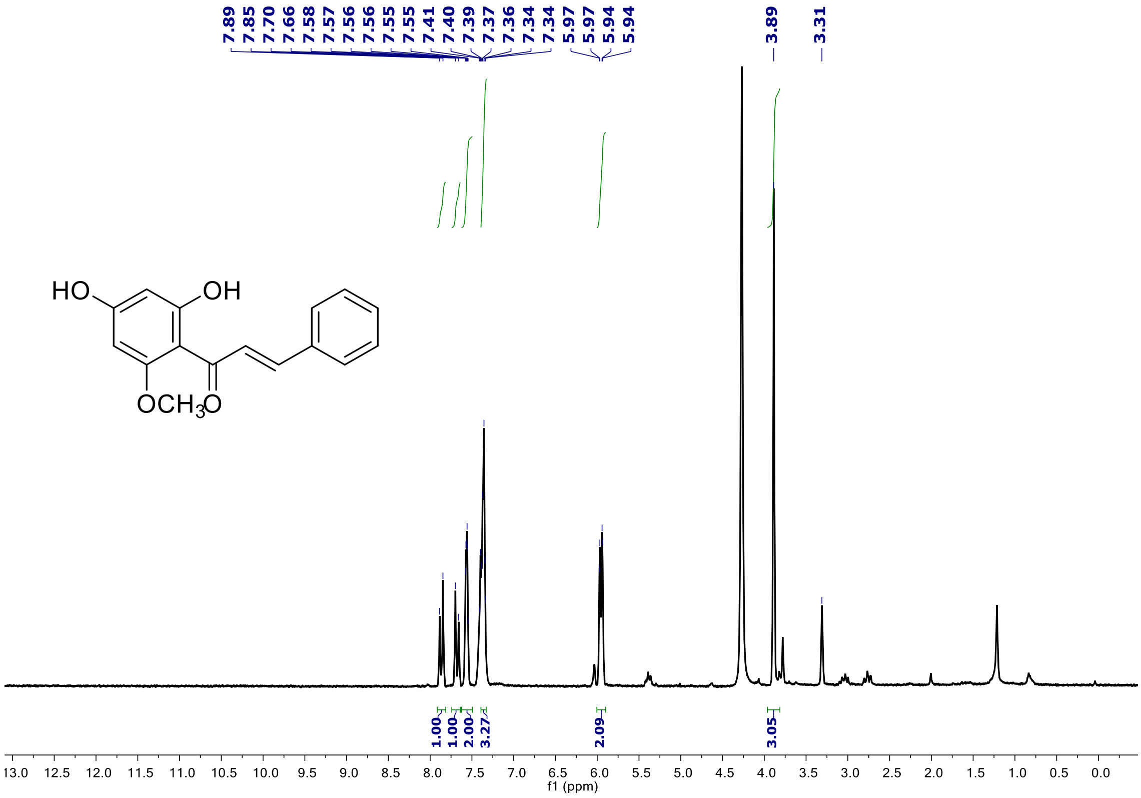

Figure S4. ^{1}H NMR spectrum (400 MHz, CD_3OD) of cardamomin (**4**)

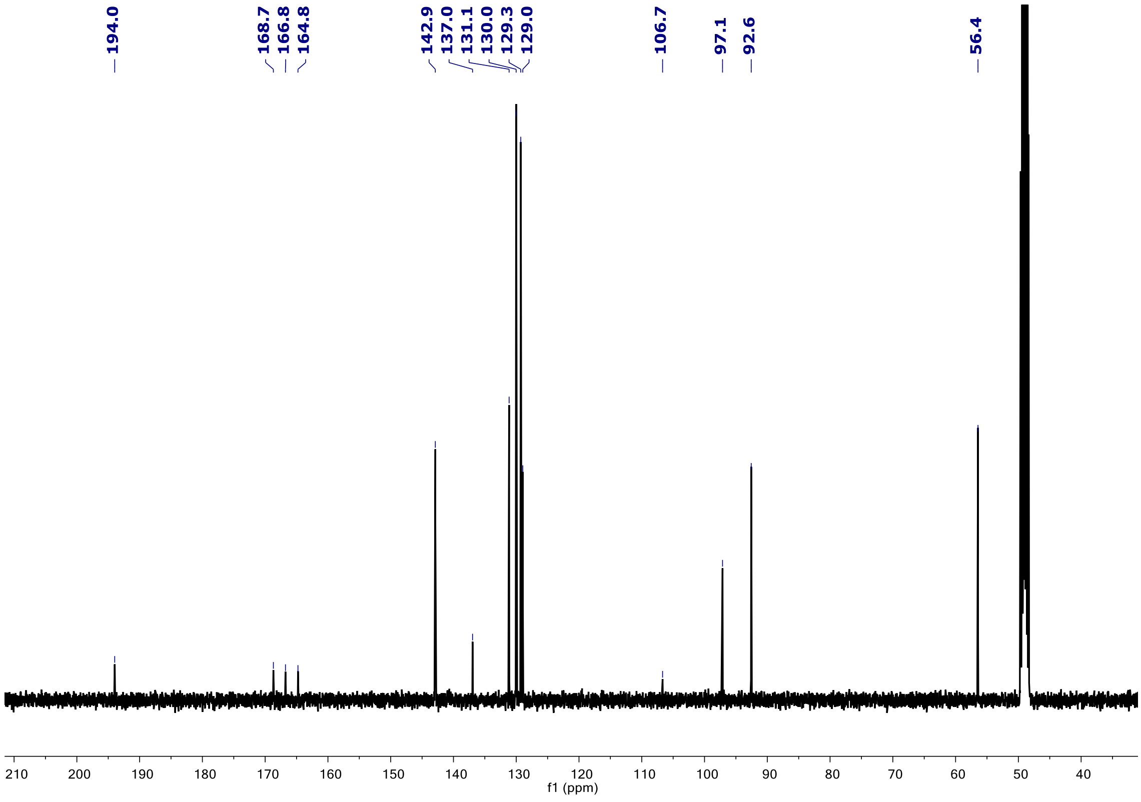

Figure S5. ^{13}C NMR spectrum (400 MHz, CD_3OD) of cardamomin (**4**)

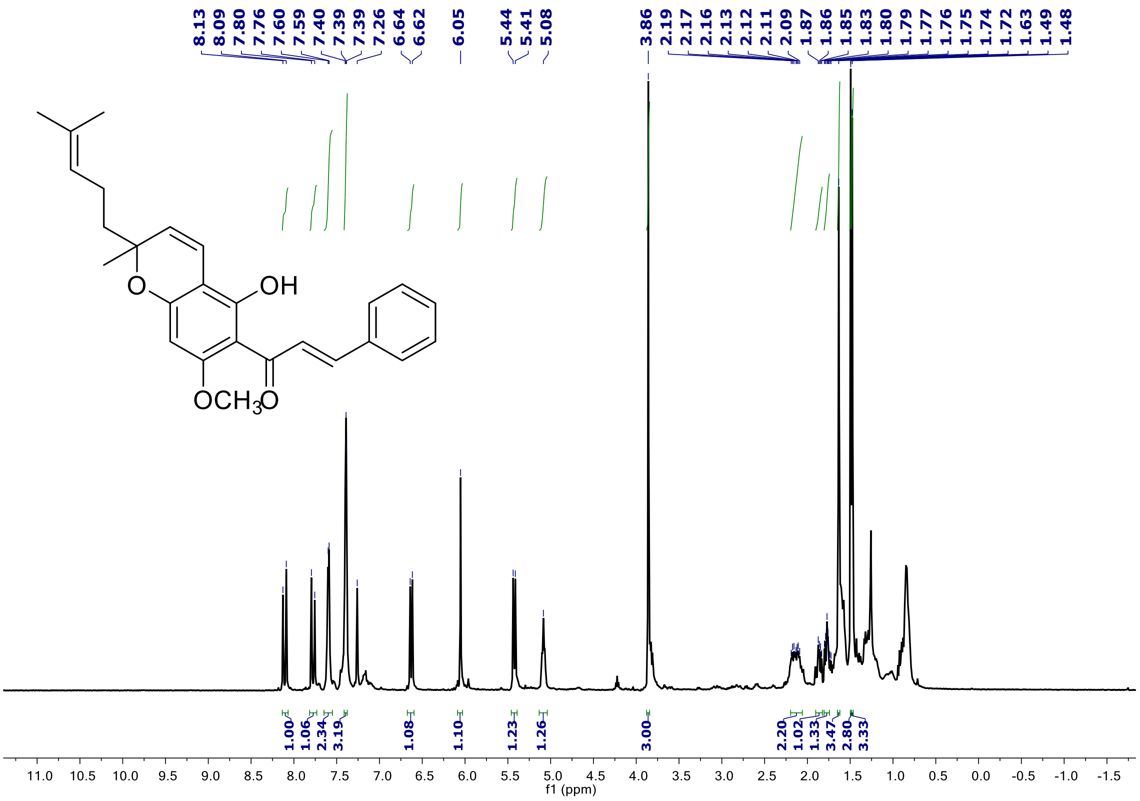

Figure S6. ^{1}H NMR spectrum (400 MHz, $CDCl_3$) of boesenbergin B (**5**)

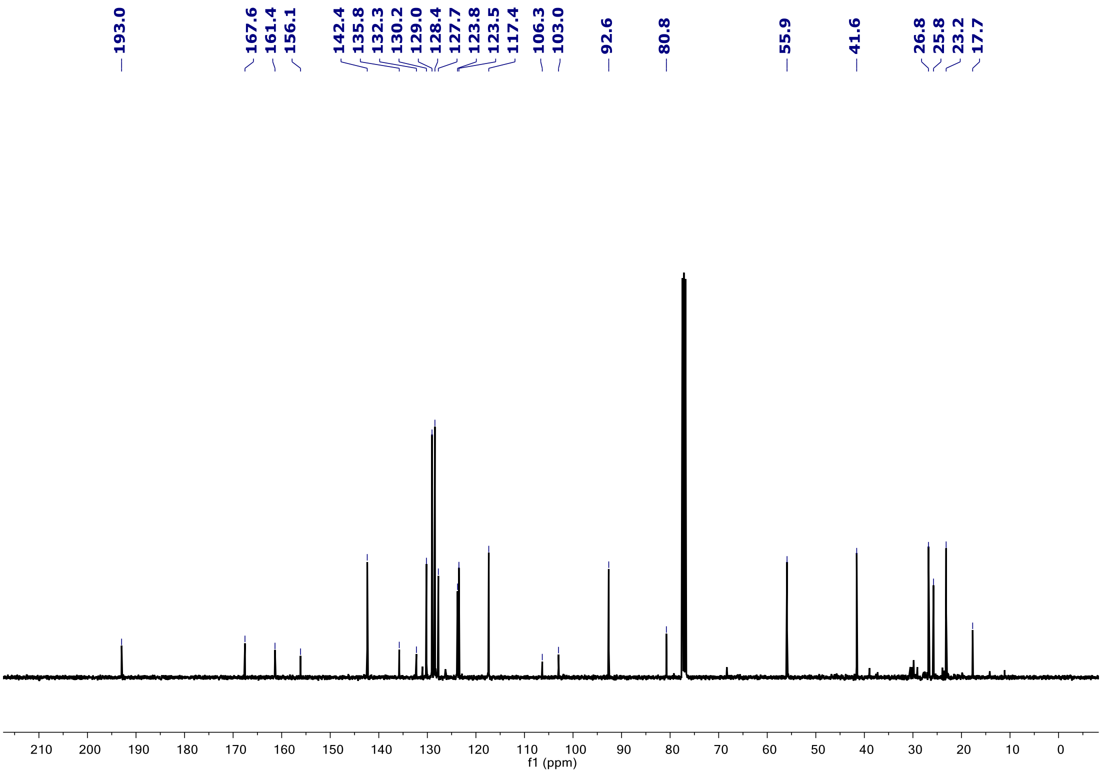

Figure S7. ^{13}C NMR spectrum (400 MHz, $CDCl_3$) of boesenbergin B (**5**)

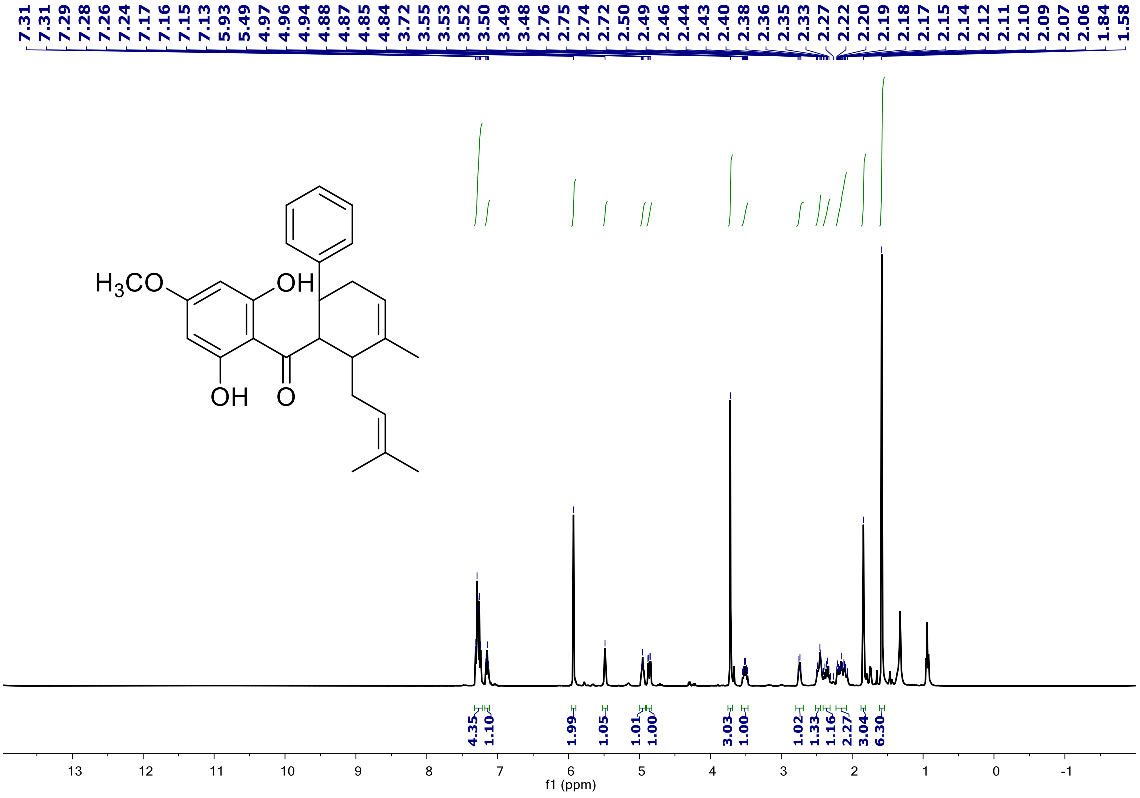

Figure S8. ^{1}H NMR spectrum (400 MHz, $CDCl_3$) of panduratin A (**6**)

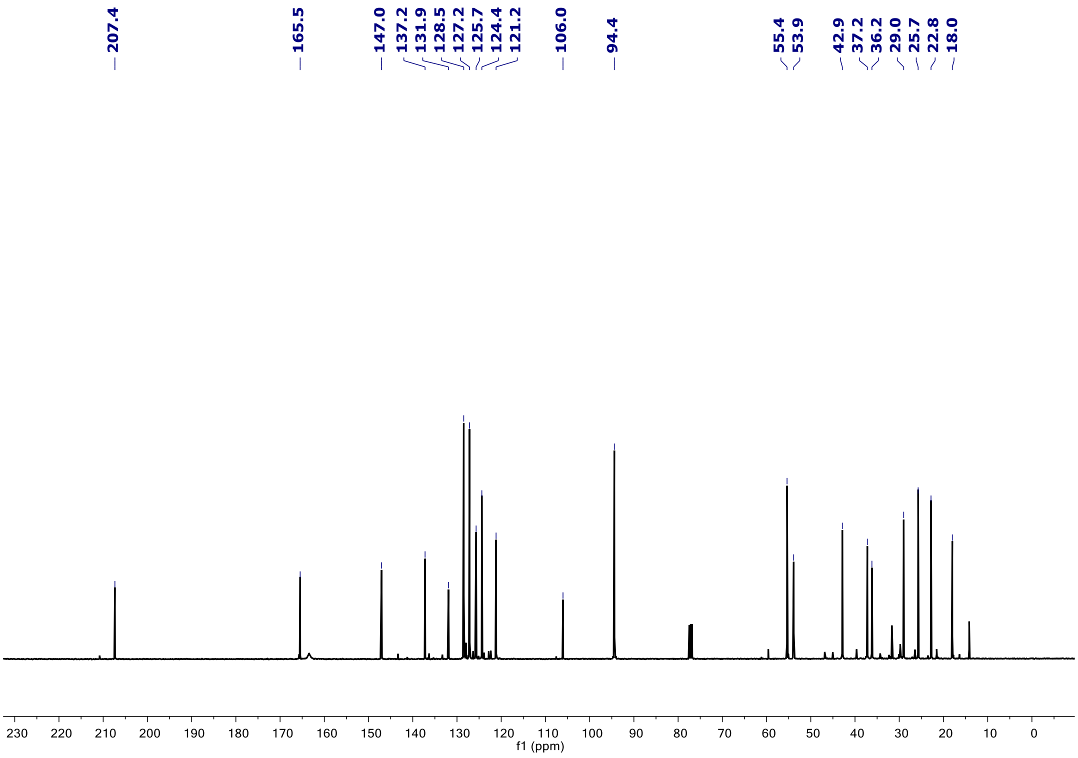

Figure S9. ^{13}C NMR spectrum (400 MHz, $CDCl_3$) of panduratin A (**6**)

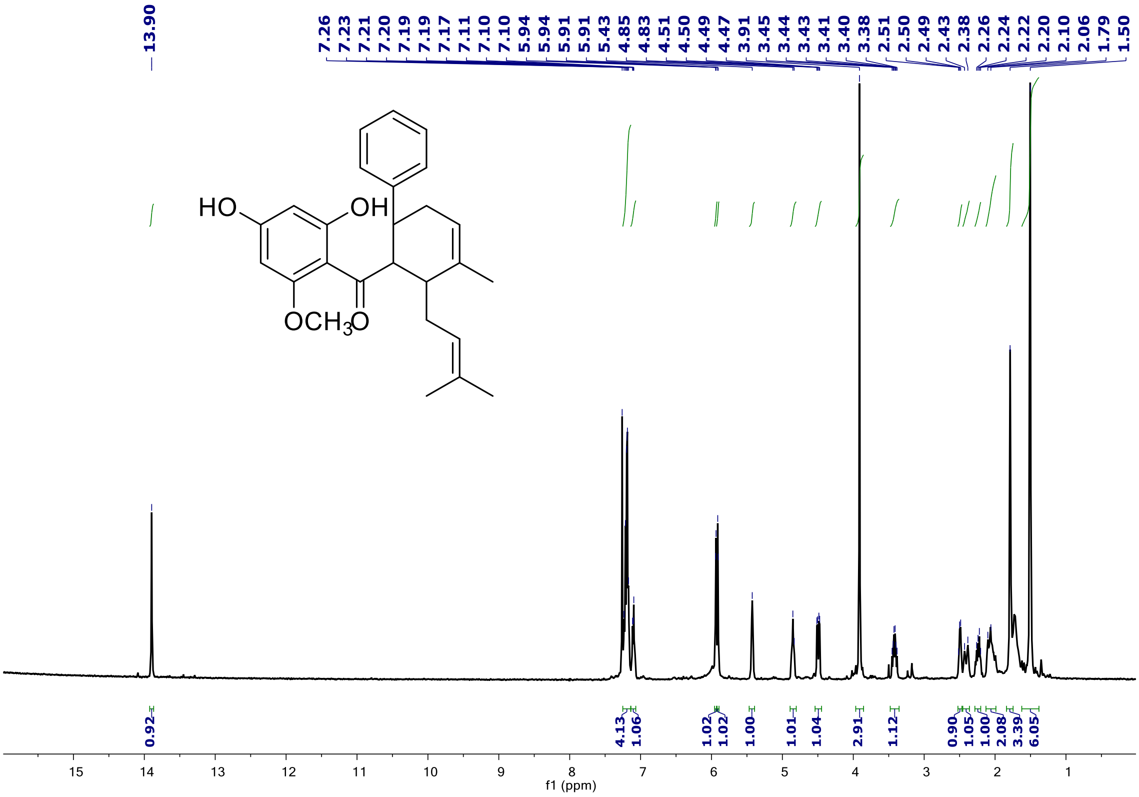

Figure S10. ^{1}H NMR spectrum (400 MHz, $CDCl_3$) of isopanduratin A (**7**)

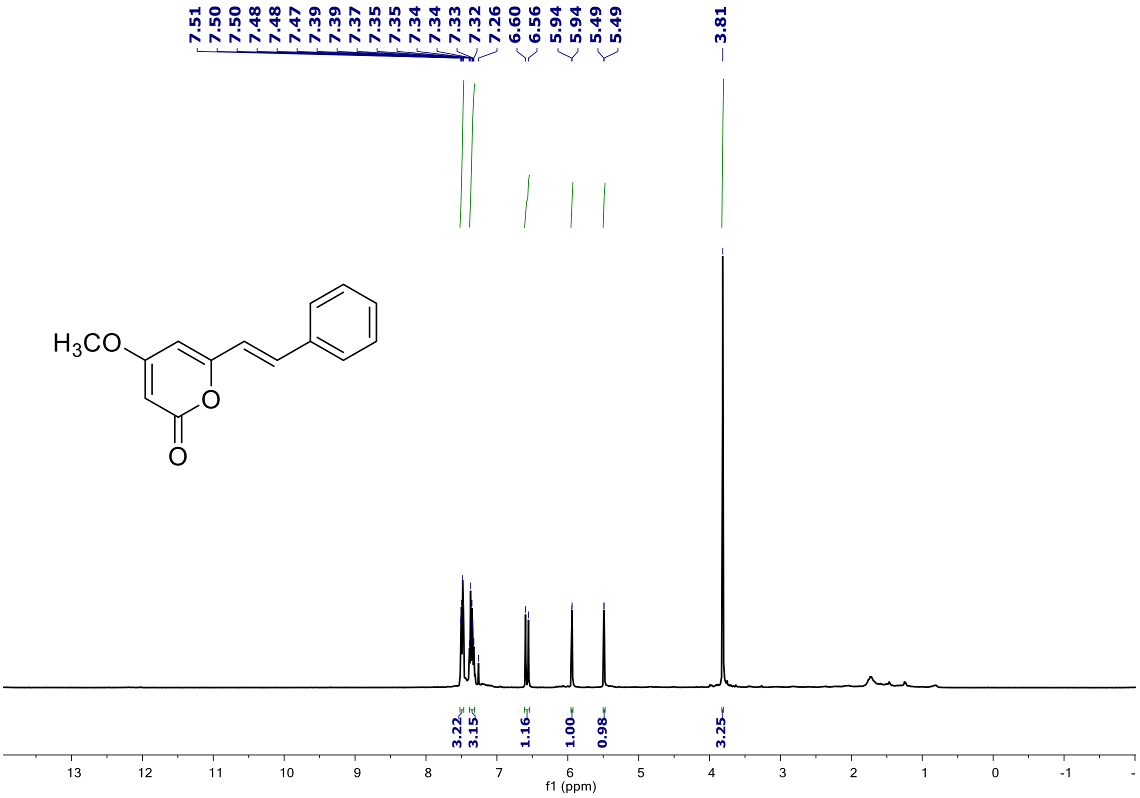

Figure S11. ^{1}H NMR spectrum (400 MHz, $CDCl_3$) of demethoxyyangonin (**8**)

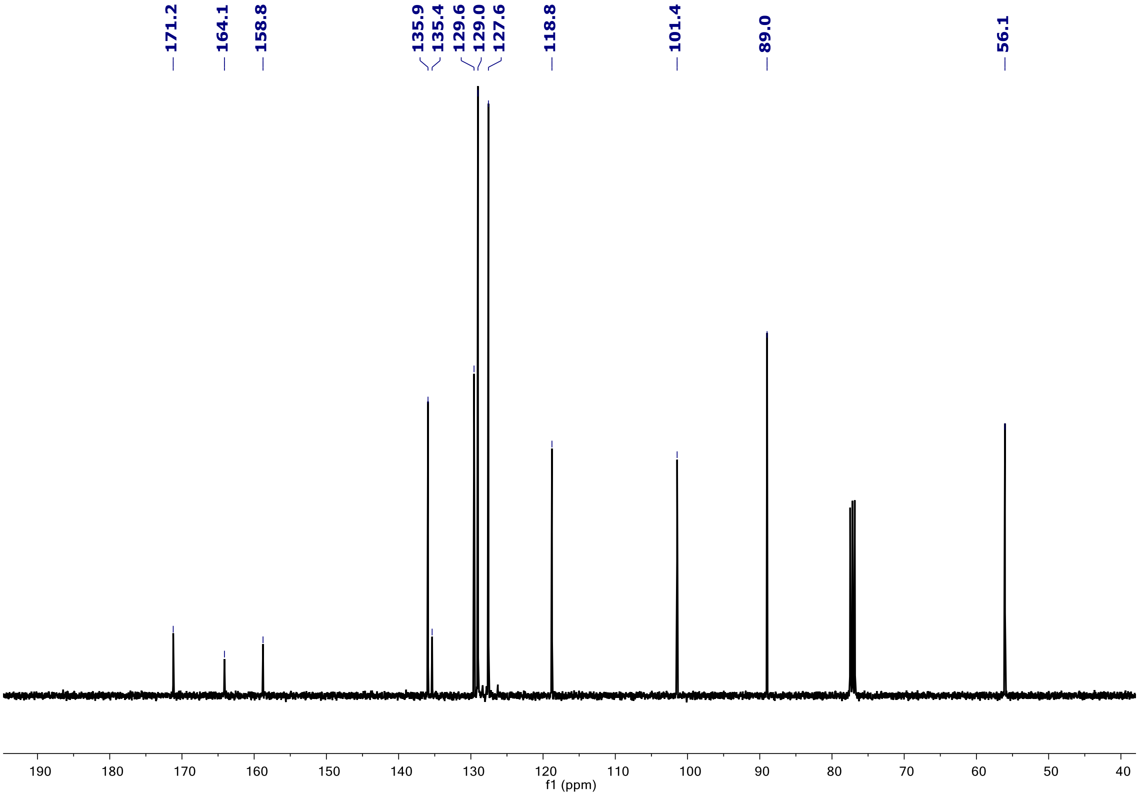

Figure S12. ^{13}C NMR spectrum (400 MHz, $CDCl_3$) of demethoxyyangonin (**8**)

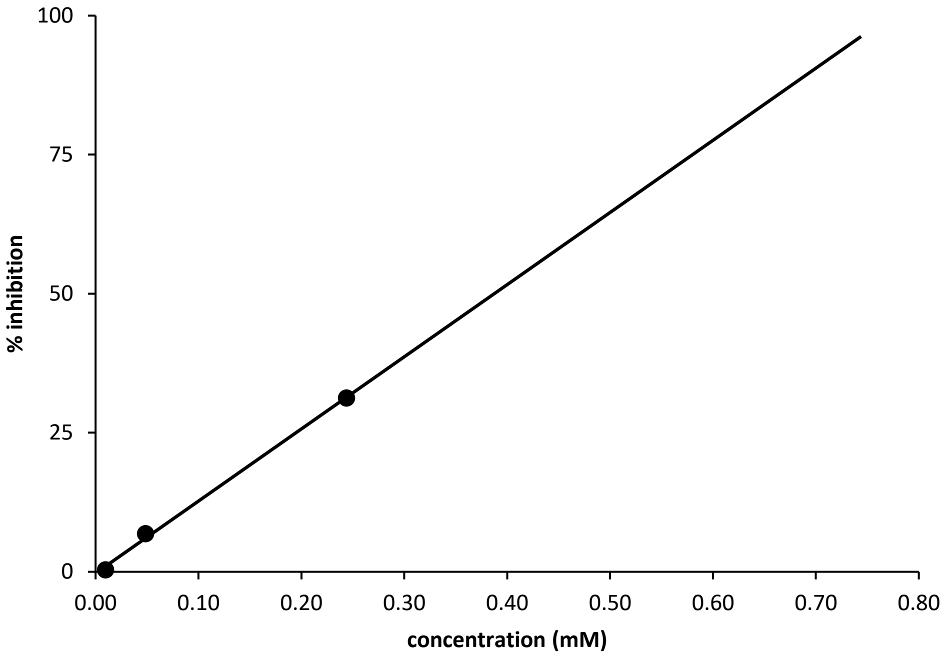

Figure S13. Inhibition plot of pinicembrin against rat intestinal maltase.

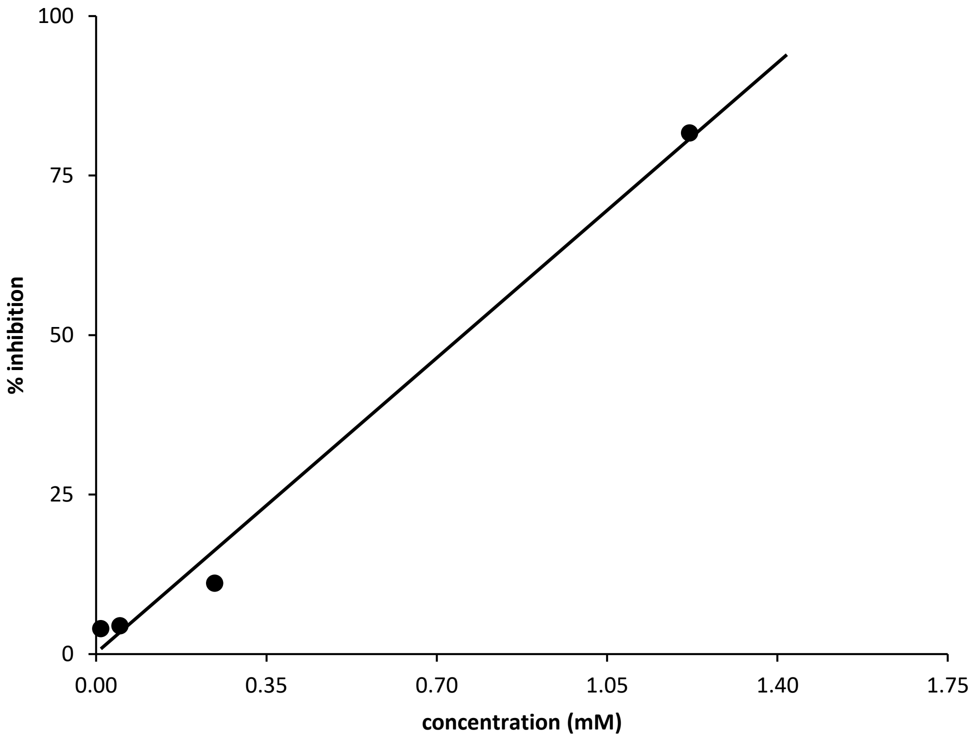

Figure S14. Inhibition plot of pinicembrin against rat intestinal sucrase.

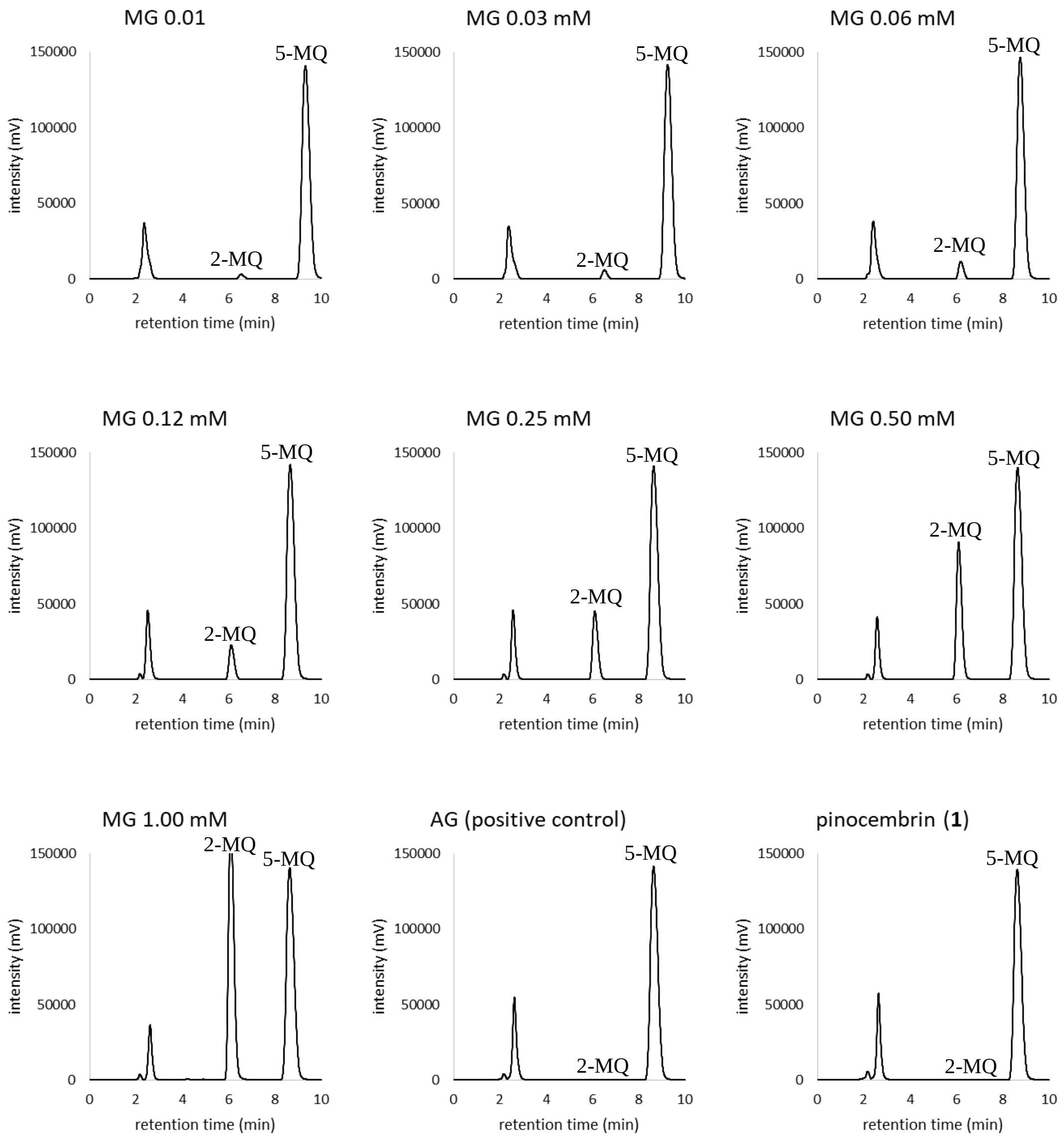

Figure S15. The HPLC chromatogram of MG (0.01–1mM) after reaction with AG (0.1 mM) and pinocembrin (0.1 mM) for 24 h. MG was detected as 2methylquinoxaline (2-MQ) after derivatization using *o*- phenylenediamine (OPD). 5-Methylquinoxaline (5-MQ) was used as the internal standard.
